# 1390. Durlobactam, a Diazabicyclooctane (DBO) β-lactamase Inhibitor (BLI), Inhibits BlaC and Peptidoglycan (PG) Transpeptidases of *Mycobacterium tuberculosis (Mtb*): A Novel Approach to Therapeutics for Tuberculosis (TB)?

**DOI:** 10.1093/ofid/ofab466.1582

**Published:** 2021-12-04

**Authors:** David Nguyen, Christopher Bethel, Magdalena A Taracilla, Qing Li, Khalid M Dousa, Sebastian G Kurz, Liem Nguyen, Barry N Kreiswirth, Wilem Boom, Robert A Bonomo

**Affiliations:** 1 University Hospitals Cleveland Medical Center/ Rainbow Babies & Children’s Hospital, Cleveland, Ohio; 2 Louis Sokes Cleveland VA Medical Center, Cleveland, OH; 3 Research Service, Louis Stokes Veterans Affairs Medical Center, Cleveland, OH; 4 Case Western Reserve University, Cleveland, Ohio; 5 Case Western Reserve University, Cleveland VA medical Center , Cleveland Heights, Ohio; 6 Mount Sinai National Jewish Health Respiratory Institute, New York City, NY; 7 Hackensack Meridian Health, Nutley, New Jersey; 8 Case Western Reserve University/ University Hospitals Cleveland Medical Center, Cleveland, Ohio; 9 Louis Stokes Cleveland VA Medical Center, Cleveland, OH

## Abstract

**Background:**

Novel therapies for multidrug-resistant TB are needed and new BLIs could answer this call. *Mtb* encodes for BlaC, a class A β-lactamase. BlaC is inhibited by clavulanate (CLA) while the DBO avibactam (AVI) is an inefficient inhibitor (low *k*_2_*/K* value). Carbapenems are hydrolyzed slowly by BlaC (low *k*_*cat*_*/K*_*m*_ value) making them “dual action” compounds that inhibit both BlaC and PG transpeptidases, the intended β-lactam targets. DBOs inhibit PG transpeptidases in other bacteria. To explore the therapeutic potential of new DBOs against *Mtb*, we compared the inhibitor activity of AVI, relebactam (REL), and durlobactam (DUR, formerly ETX2514) against BlaC and *Mtb* PG transpeptidases using a biochemical approach. We also investigated the ability of DUR to lower minimum inhibitory concentrations (MICs) of β-lactams against *Mtb* H37Rv.

**Methods:**

Mass spectrometry was performed to capture acyl-enzyme complexes (AECs) of purified BlaC and PG transpeptidases (PonA1, Ldt_Mt1_, Ldt_Mt2,_ Ldt_Mt3_, and Ldt_Mt5_) with β-lactams and BLIs. Steady-state enzyme kinetics were determined using nitrocefin as a substrate. MICs with amoxicillin (AMX), meropenem (MER), CLA, and DUR alone and in combination against *Mtb* H37Rv were assessed using a microdilution method.

**Results:**

DUR alone had a MIC of 2 µg/mL with *Mtb* H37Rv (Table 1). BlaC formed AECs with all carbapenems and BLIs. BlaC had lower *K*_*i* app_ and higher *k*_*2*_/*K* with DUR than those with AVI and REL and comparable to those with CLA; however, with a period of pre-incubation, AVI fully inhibits BlaC (Table 2). The carbapenems and DUR formed the most AECs with PG transpeptidases of the β-lactams and BLIs respectively; PG transpeptidases had lower *K*_*i* app_ values with DUR than those with AVI (Table 3).

Table 1. Minimum Inhibitory Concetrations for Mycobacterium tuberculosis H37Rv

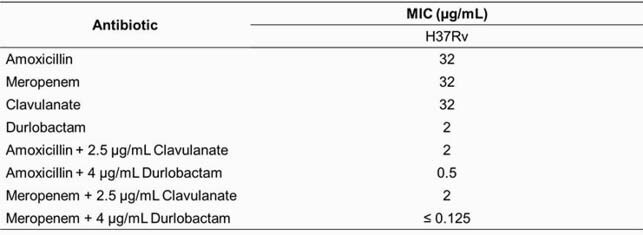

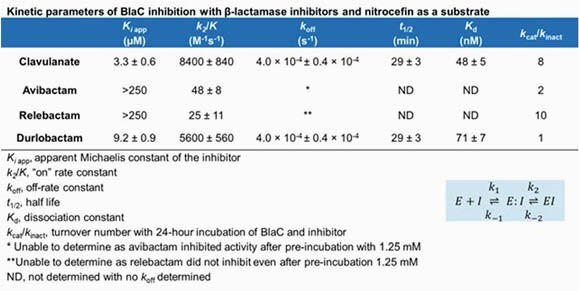

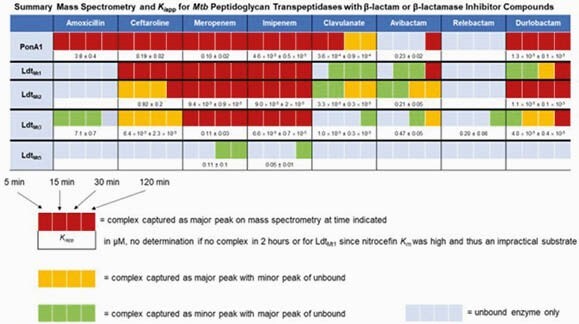

**Conclusion:**

DUR alone has some antimicrobial activity against *Mtb* H37Rv. The likely mechanism that underlies this activity is inhibition of BlaC and several PG transpeptidases. Inhibition of enzyme targets with DUR was more potent and efficient than AVI and REL. DUR in combination with β-lactams lowered MICs but the DUR concentration used was higher than its MIC. Our findings support the exploration of novel BLIs against BlaC and PG transpeptidases with the ultimate goal of repurposing these drugs for the treatment of TB.

**Disclosures:**

**Robert A. Bonomo, MD**, **entasis** (Research Grant or Support)**Merck** (Grant/Research Support)**NIH** (Grant/Research Support)**VA Merit Award** (Grant/Research Support)**VenatoRx** (Grant/Research Support)

